# A novel role for 3, 4-dichloropropionanilide (DCPA) in the inhibition of prostate cancer cell migration, proliferation, and hypoxia-inducible factor 1alpha expression

**DOI:** 10.1186/1471-2407-6-204

**Published:** 2006-08-02

**Authors:** Bing-Hua Jiang, Ling-Zhi Liu, Rosana Schafer, Daniel C Flynn, John B Barnett

**Affiliations:** 1 Department of Microbiology, Immunology, and Cell Biology, West Virginia University, Morgantown, West Virginia 26506, USA; 2 The Mary Babb Randolph Cancer Center, West Virginia University, Morgantown, West Virginia 26506, USA

## Abstract

**Background:**

The amide class compound, 3, 4-dichloropropionanilide (DCPA) is known to affect multiple signaling pathways in lymphocyte and macrophage including the inhibition of NF-κB ability. However, little is known about the effect of DCPA in cancer cells. Hypoxia-inducible factor 1 (HIF-1) regulates the expression of many genes including vascular endothelial growth factor (VEGF), heme oxygenase 1, inducible nitric oxide synthase, aldolase, enolase, and lactate dehydrogenase A. HIF-1 expression is associated with tumorigenesis and angiogenesis.

**Methods:**

We used Transwell assay to study cell migration, and used immunoblotting to study specific protein expression in the cells.

**Results:**

In this report, we demonstrate that DCPA inhibited the migration and proliferation of DU145 and PC-3 prostate cancer cells induced by serum, insulin, and insulin-like growth factor I (IGF-I). We found that DCPA inhibited HIF-1 expression in a subunit-specific manner in these cancer cell lines induced by serum and growth factors, and decreased HIF-1α expression by affecting its protein stability.

**Conclusion:**

DCPA can inhibit prostate cancer cell migration, proliferation, and HIF-1α expression, suggesting that DCPA could be potentially used for therapeutic purpose for prostate cancer in the future.

## Background

The genetic alterations in human cancer are a major focus of cancer research over the past two decades. Many genetic alterations such as activation of oncogenes and inactivation of tumor suppression genes lead to the increased expression of Hypoxia-inducible factor 1 (HIF-1) [1-4]. HIF-1 is a heterodimeric transcription factor composed of HIF-1α and HIF-1β subunits [5,6]. HIF-1 regulates the expression of many genes including vascular endothelial growth factor (VEGF), heme oxygenase 1, inducible nitric oxide synthase, aldolase, enolase, and lactate dehydrogenase A [7]. HIF-1 activity correlates with tumorigenesis and angiogenesis when wild type and HIF-1-deficient cell lines levels were injected into nude mice [1,8]. HIF-1 is overexpressed in many human cancers including prostate cancer [9]. HIF-1 expression in prostate cancer cells is induced by growth factors, and inhibited by phosphatidylinositol 3-kinase (PI3K) inhibitors and the tumor suppressor PTEN [2,10].

The amide class compound, DCPA is a dichlorinated ring compound of low molecular weight. Previous investigations on the effects of this compound on lymphocyte and macrophage signaling pathways, reveal that this compound reduces NF-κB DNA binding ability [11]. This correlates with the down regulation of the production of a number of proinflammatory cytokines, including tumor necrosis factor-α, interleukin (IL)-6, IL-1β [11-13] as well as IL-2 [14-17] DCPA also inhibits activation of key components of the Ras pathway [17]. The noted down regulation of the signaling pathways leads us to speculate that DCPA may also affect these pathways in cancer cells in a manner that could be exploited to provide a mode of chemotherapy against these tumors. In this study, we show that DCPA inhibited serum and growth factor-induced cell migration and proliferation, and inhibited HIF-1 expression induced by serum and growth factors in prostate cancer cells. This study indicates that DCPA or its analog may represent a new therapeutic agent for cancer treatment due to its relatively low toxicity.

## Methods

### Cell culture

The human prostate cancer cell line, DU145, was cultured in minimum essential medium (MEM) with Earle's salts and glutamine (Gibco BRL, Grant Island, NY), supplemented with 10% fetal bovine serum (FBS) (Hyclone) and 3% chicken serum (Gibco BRL, Grant Island, NY). The human prostate cancer cell line, PC-3, was cultured in F-12K nutrient mixture (Kaighn's modification) (Gibco BRL), supplemented with 10% FBS and 1% chicken serum. The cells were incubated at 37°C in 5% CO_2 _incubator.

### Cell migration assay

The DU145 and PC-3 cells were cultured in normal medium overnight, then switched to serum-free MEM medium in the presence or absence of DCPA for 18 h. The cells were harvested in Hanks balanced salt solution with 5 mM EDTA and 25 mM HEPES, pH 7.2, washed, and resuspended in serum-free medium (SFM). Cell migration was assayed using a polystyrene Transwell plate (Corning Costar, Cambridge, MA) with 6.5 mm diameter wells and pore sizes of 8.0 μm. The wells were coated with 10 μg/ml collagen type 1 (Upstate Biotechnology, Lake Placid, NY) in 1× PBS buffer at 4°C overnight. The excess collagen was removed from the bottom chambers after the incubation, and replaced with 250 μl serum-free medium in the absence or presence of DCPA (150 μM) or 10% FBS. The cells were counted and diluted to 400,000 cells per ml in SFM, and 250 μl of the cells were added to the top of each well. For the DCPA treatment, the cells were incubated with SFM with 150 μM DCPA 20 min prior to the addition of 100,000 cells to each well in the presence or absence of 10% FBS in the bottom chambers, followed by the addition of 10% serum, 200 nM insulin, or 2 nM insulin-like growth factor I (IGF-I) for 6 h. The wells were incubated at 37°C in 5% CO_2_incubator for 6 h. The wells were removed, and the cells on the top of the well were wiped out with a cotton swab, stained with 1% crystal violet in 0.1 M borate containing 2% ethanol, pH 9.0 for 20 min. The migrated DU145 and PC-3 cells were rinsed with water, dried, and counted using light microscope with 10× magnification.

### Cell viability assay

The DU145 and PC-3 cells were seeded at 5 × 10^5 ^cells per well in a 24-well plate, and incubated at 37°C in 5% CO_2 _incubator in the complete medium for 24 h. Then, the culture medium was removed and replaced with serum-free medium containing DCPA (25, 75 or 150 μM) or solvent alone (ETOH). A medium-only control was also included. After the culture in serum-free medium for 24 h, the cells were switched to the medium with 10% serum for 6 h. The cells were then stained with trypan blue, and counted using standard hematocytometer methods.

### Cell proliferation assay

One day before the assay, DU145 and PC-3 cells were seeded at 100,000 cells per well in a six-well plate and incubated at 37°C in 5% CO_2 _incubator in the medium as described above. Then the cells were switched to fresh medium containing DCPA (150 μM) or solvent alone (0 μM DCPA), and the cell number was counted 24, 48, and 72 h after the treatment using standard hematocytometer methods.

### Protein extraction and immunoblotting

DU145 and PC-3 cells were harvested in cold 1× PBS and lysed on ice for 30 min in RIPA buffer (150 mM NaCl, 100 mM Tris, pH 8, 0.1% SDS, 1% Triton X-100, 1% sodium deoxycholate, 5 mM EDTA and 10 mM NaF) supplemented with 1 mM sodium vanadate, 2 mM leupeptin, 2 mM aprotinin, 1 mM phenylmethylsulfonyl fluoride (PMSF), 1 mM DTT, and 2 mM pepstatin A. The lysates were cleared by centrifugation at 14,000 rpm for 15 min, and the supernatants were collected as total cellular protein extracts. The protein concentration in the extracts was determined using Bio-Rad protein assay reagent (Bio-Rad, Richmond, CA). The protein extracts were separated by SDS-polyacrylamide gel electrophoresis (SDS-PAGE), and transferred to nitrocellulose membranes in 20 mM Tris-HCl (pH 8.0) containing 150 mM glycine and 20% (v/v) methanol. Membranes were blocked with 5% nonfat dry milk in 1× TBS containing 0.05% Tween 20 and incubated with antibodies against HIF-1α and HIF-1β [18]. The protein bands were detected by the incubation with horseradish peroxidase-conjugated antibodies (NEN, Boston, MA), and visualized using the enhanced chemiluminescence reagent (NEN).

### HIF-1α protein stability analysis

PC-3 cells were cultured in serum-free medium for 24 h, followed by the incubation with medium containing 10% fetal bovine serum for 5 h. Then the cells were treated with solvent alone or 150 μM DCPA for 1 h, followed by the incubation with 100 μM cycloheximide for 0 to 16 min. Cellular lysates were subjected to immunoblotting using antibodies against HIF-1α and HIF-1β. The intensity of HIF-1α/HIF-1β protein signals was quantified using EagleSight densitometry software (Version 3.21; Stratagene).

### Statistical analysis

The results on DU145 and PC-3 cell migration and proliferation were analyzed statistically using the Student-Newman-Keuls multicomparisons test using SigmaStat Software (SPSS Inc., Chicago, IL).

## Results and discussion

### DCPA inhibits prostate cancer cell migration

**Figure 1:**
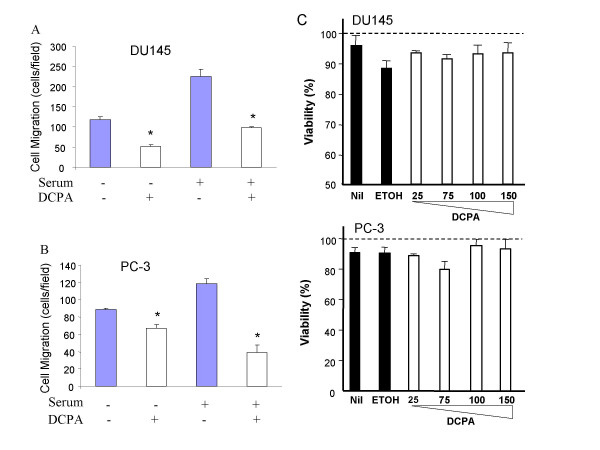
Migration of prostate cancer cells was inhibited by DCPA. Prostate cancer cells, DU145 (**A**) and PC-3 (**B**) cells, were cultured in serum-free medium in the presence (+) or absence (-) of 150 μM DCPA for 18 h as indicated. The cell migration assay was performed using Transwell migration chambers coated with 10 μg/ml collagen in the migration buffer with or without 10% fetal bovine serum for 6 h at 37°C as described in Materials and Methods. The migrated cells were counted, and data were expressed as mean of cell counts from replicate experiments. Bars, SD; * indicates significant difference when compared to cell migration without DCPA (P < 0.01). **C**. Viability of the prostate cancer cells was not affected by DCPA. The cells were cultured in the absence or presence of DCPA for 24 h, and cell viability assay was performed as described in Materials and Methods. The results were expressed as the mean percentage of cells capable of excluding trypan blue. These experiments were repeated at least twice. Bars, SD.

The hallmark of tumor cells is their ability to migrate and metastasize. Prostate cancers are known to metastasize in a high percentage of the cases, which is obviously linked to a poor prognosis. Therefore, an agent that could inhibit cell migration with relatively low toxicity would be of benefit, likely as adjunct therapy to more traditional cancer drugs. To determine a potential effect of DCPA on cancer cell migration, cell migration on a collagen substrate was tested using the prostate cancer cell lines, DU145 and PC-3. The results demonstrated that the migration of DU145 cells in medium with serum has two-fold greater than that in serum-free medium (Fig. 1A). DCPA treatment inhibits the cell migration more than two-fold as compared to the solvent control in the presence or absence of serum (Fig. 1A). In PC-3 cells, serum increased the cell migration by 50% more than the serum-starved cells (Fig. 1B). Similarly, DCPA inhibited the cell migration in the presence and absence of serum. These results suggest that DCPA can inhibit the basal-level and serum-induced cell migration.

To determine if the decreased cell migration was due to decreased cell viability, cell viabilities were determine on separate cultures treated as described above. As shown in Fig. 1C, neither cell line showed any increase in cell death over either the solvent (ETOH) or medium (nil) controls. Thus, the inhibition of cell migration by DCPA is due to the effect of DCPA on the signaling pathways associated with cell motility and not simply due to increased cell death.

### DCPA inhibits cell proliferation

**Figure 2:**
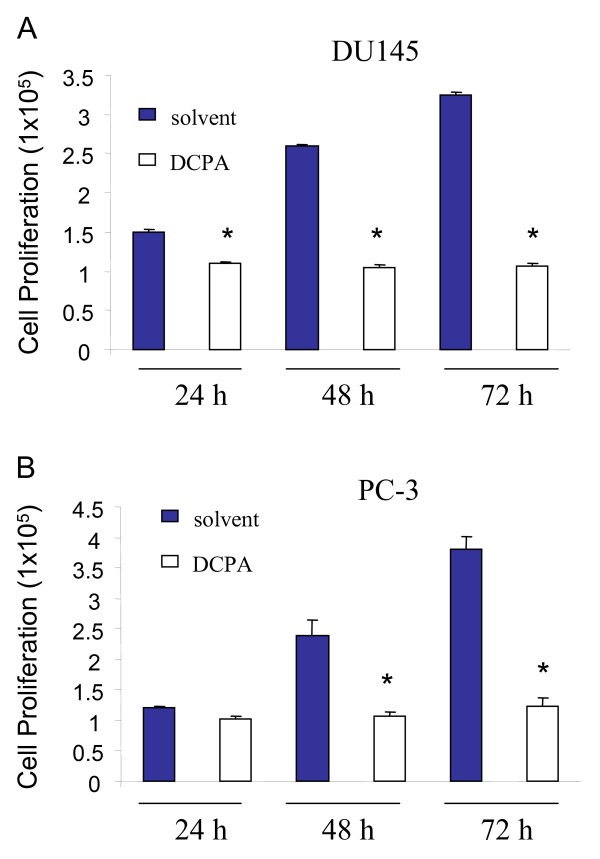
Proliferation of prostate cancer cells was inhibited by DCPA. DU145 (**A**) and PC-3 (**B**) cells were seeded at 100,000 cells per well one day before the addition of DCPA. The cells were incubated in the presence (+) or absence (-) of 150 μM DCPA for 24, 48, and 72 h, as indicated. The cell proliferation assay was performed as described in Materials and Methods. The results are expressed as mean of cell number from replicate experiments. Bars, SD. * indicates significant difference when compared to cell proliferation without DCPA at the same time point (P < 0.01).

Unchecked proliferation is also a hallmark of cancer cells that commonly exhibit increased proliferation when compared to normal cells. To examine the effect of DCPA on the proliferation of DU145 and PC-3 cells, we determined the cell proliferation after the treatment for 24, 48, and 72 h. The cell number at 24 h culture was used as a baseline. In the solvent control, the number of DU145 cells increased by 50% and 100% at 48 h and 72 h, respectively. However, cells cultured in the presence of DCPA did not increase significantly throughout the 72 h culture period (Fig. 2A). Similarly, DCPA treatment inhibited the proliferation of PC-3 cells (Fig. 2B). PC-3 cells cultured in the presence of solvent control showed 3-fold increase in cell number over the 72 h period. However, PC-3 cells cultured in the presence of DCAP showed no net change in cell numbers over the 72 h period (Fig. 2B). These results suggest that DCPA treatment is sufficient to inhibit the cell proliferation.

### DCPA treatment diminishes HIF-1α expression induced by serum

**Figure 3:**
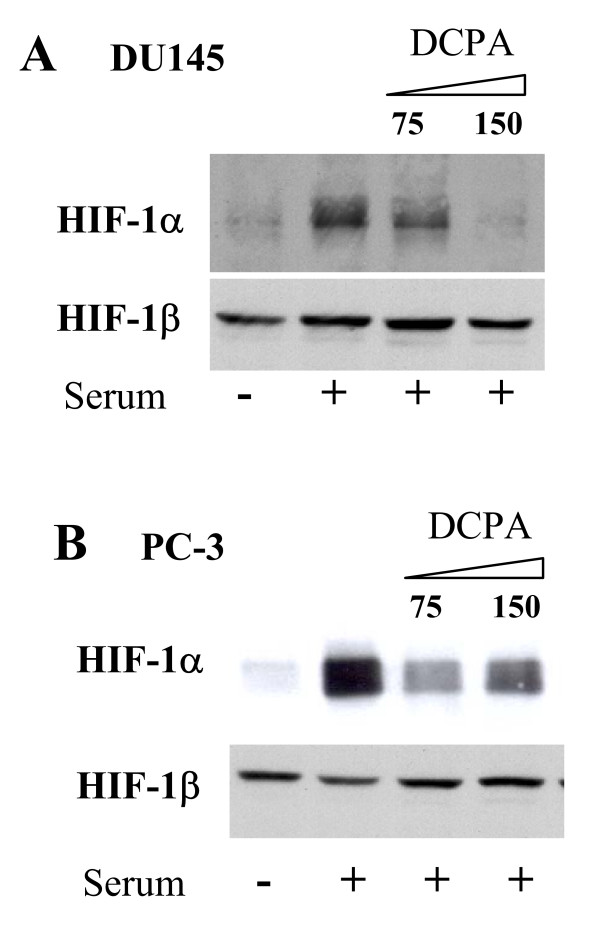
Expression of HIF-1α was specifically induced by the addition of serum, and inhibited by the presence of DCPA. DU145 (**A**) and PC-3 (**B**) cells were cultured in serum-free MEM basal medium with Earle's salt in 0, 75, or 150 μM DCPA for 24 h, followed by the incubation with or without 10% fetal bovine serum for 6 h. Total cellular protein extracts were prepared from these cells, and aliquots of the extracts (30 μg) were used for the immunoblotting analysis using antibodies against HIF-1α and HIF-1β.

We previously showed that activation of oncogenes, v-Src and PI3K, increased HIF-1 expression [1,2]. HIF-1 is well established as a transcriptional regulator of VEGF and many other genes involved in tumor growth and angiogenesis [1,7,8]. HIF-1α expression is increased in prostate cancer cells by the addition of serum [2,10]. We sought, therefore, to determine whether DCPA affected HIF-1 expression induced by serum. DU145 and PC-3 cells were cultured without serum for 18 h in the absence or presence of DCPA, then exposed to 10% serum for 6 h. Serum significantly induced the expression of HIF-1α in DU145 cells (Fig. 3A), and in PC-3 cells (Fig. 3B). Serum did not increase the levels of HIF-1β expression (Fig. 3). The addition of DCPA decreased the induction of HIF-1α expression by serum. The levels of HIF-1α expression were diminished to below basal values by 150 μM DCPA in DU145 cells (Fig. 3A). The levels of HIF-1β expression were not affected by DCPA. This study indicated that DCPA specifically inhibited HIF-1α expression induced by serum.

**Figure 4:**
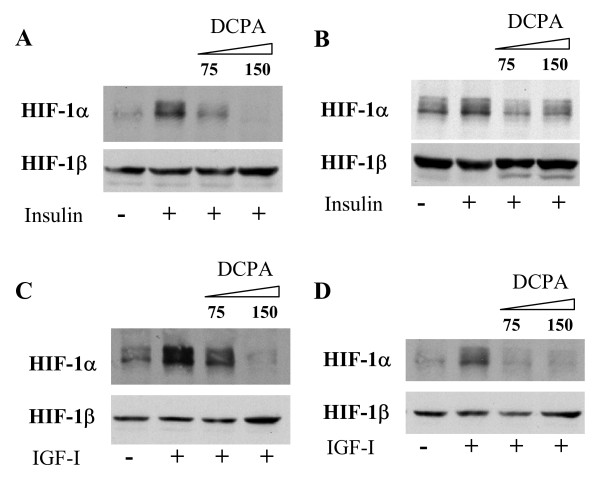
DCPA specifically inhibits expression of HIF-1α induced by insulin and IGF-I. DU145 (**A**) and PC-3 (**B**) cells were cultured in the basal medium without serum in the presence of 0, 75 or 150 μM DCPA for 24 h, followed by the incubation with (+) or without (-) 200 nM insulin for 6 h. The total cellular proteins were prepared from the cells, and used for the analysis of HIF-1α and HIF-1β protein expression as described above. DU145 (**C**) and PC-3 (**D**) cells were cultured in the basal medium without serum as above, followed by the incubation in the presence (+) or absence (-) of 2 nM IGF-I for 6 h. The HIF-1α and HIF-1β protein expression from the cells was analyzed by immunoblotting.

HIF-1α expression is also up-regulated in response to growth factor signaling [2,10]. In order to determine whether DCPA could impede growth factor mediated up-regulation of HIF-1α expression, cells were induced with insulin and IGF-I in the presence or absence of DCPA. The DU145 and PC-3 cells were cultured in the absence of serum for 18 h, followed by the exposure of insulin or IGF-I for 6 h. HIF-1α expression in DU145 cells was significantly induced by insulin and IGF-I, and the induced HIF-1α expression was inhibited by DCPA (Figs. 4A and 4C). But HIF-1β expression was not affected by the addition of insulin and IGF-I or by the presence of DCPA (Figs. 4A and 4C). We also analyzed PC-3 cells for the comparison. HIF-1α expression in PC-3 cells was also greatly induced by both insulin and IGF-I, and inhibited by the addition of DCPA (Figs. 4B and 4D). In PC-3 cells, the induction of HIF-1α expression by IGF-I was greater than that by insulin (Figs. 4B and 4D). The expression of HIF-1β was not affected in the same experiment. These results suggest that DCPA affects HIF-1 expression in a subunit-specific manner in prostate cancer cells, inhibits HIF-1α expression induced by growth factors such as insulin and IGF-I, and further suggest that DCPA may be a potential specific molecule to target the growth factor/HIF-1 pathway for cancer therapy.

**Figure 5:**
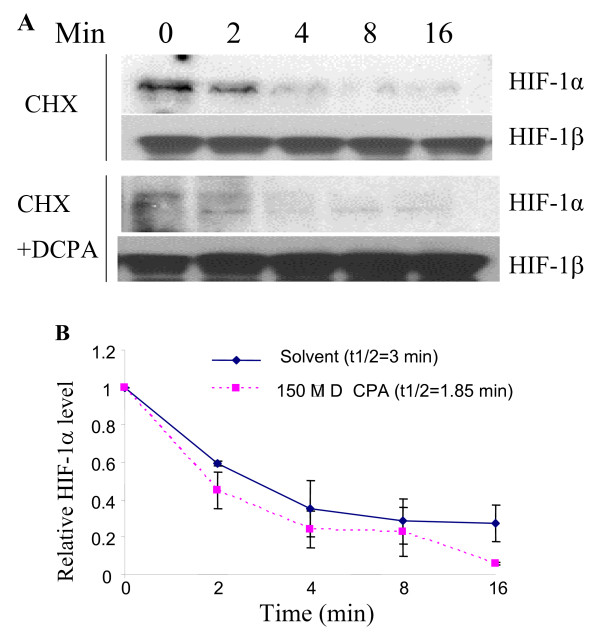
Effect of DCPA on HIF-1α protein stability. **A**. PC-3 cells were cultured in serum-free medium for 24 h, followed by the incubation with 10% fetal bovine serum for 5 h. Then the cells were treated with solvent alone (CHX, top panel) or 150 μM DCPA (CHX+DCPA, bottom panel) for 1 h, followed by the incubation with 100 μM cycloheximide for 0 to 16 min. Cellular lysates were subjected to immunoblotting using antibodies against HIF-1α and HIF-1β. **B**. The intensity of HIF-1α protein signals obtained above was quantified from three replicate experiments using EagleSight densitometry software (Version 3.21; Stratagene). The densitometry data were the ratio of HIF-1α/HIF-1β, and normalized to that of the control (0 min).

**Figure 6:**
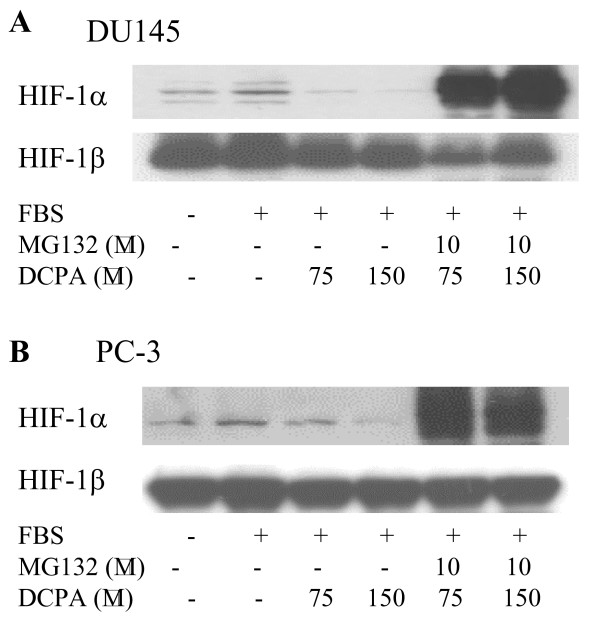
MG132 treatment prevented the inhibition of HIF-1α expression by DCPA. DU145 (**A**) and PC-3 (**B**) cells were cultured in serum-free MEM basal medium with Earle's salt in the presence of 0, 75, or 150 μM DCPA for 24 h, followed by the treatment with or without 10 μM of MG132 for 30 min. Cells were incubated with or without 10% fetal bovine serum for 6 h. Total cellular protein extracts were prepared from these cells, and aliquots of the extracts (30 μg) were used for the immunoblotting analysis using antibodies against HIF-1α and HIF-1β.

**Figure 7:**
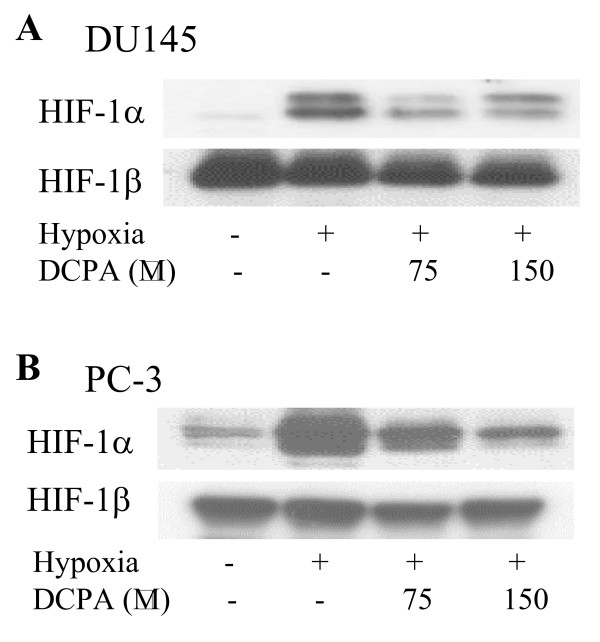
Expression of HIF-1α was specifically induced by hypoxia, and inhibited by the treatment of DCPA. DU145 (**A**) and PC-3 (**B**) cells were cultured in complete medium in the presence of 0, 75, or 150 μM DCPA for 24 h, followed by the incubation with 20% or 1% O_2 _(hypoxia) for 6 h. Total cellular protein extracts were prepared from these cells, and aliquots of the extracts were used for the immunoblotting analysis.

We demonstrated that DCPA specifically inhibits HIF-1α, but not HIF-1β expression in prostate cancer cells. DCPA may regulate HIF-1α expression by altering either the stability of HIF-1α protein, or by inhibiting its mRNA levels. HIF-1α protein is known to interact with tumor suppressors p53 and von Hippel-Lindau (VHL) protein [19]. Under normal oxygen tension, HIF-1α is degraded by proteasome degradation pathway. Proteasomal degradation is likewise regulated by p53 and VHL, which serve to increase the efficiency of ubiquination of HIF-1α. Conversely, ubiquination of HIF-1α is decreased by hypoxia, a condition commonly observed in tumors [20, 21]. To determine whether DCPA treatment affected HIF-1α protein stability, PC-3 cells were treated with or without DCPA in the presence of 100 μM cycloheximide, which inhibits new protein synthesis in the cells. HIF-1α protein stability was analyzed in the cells. DCPA treatment greatly decreased the HIF-1α protein stability with 40% reduction of HIF-1α protein half-life in the cells (Fig. 5). DCPA treatment did not affect HIF-1α mRNA level in the cells (data not shown). This result suggests that DCPA treatment may inhibit HIF-1α protein stability through the proteasomal degradation pathway. To further confirm this hypothesis, we treated the cells with DCPA in the absence or presence of proteasome inhibitor MG132. The presence of MG132 greatly increased HIF-1α protein levels to much higher levels even in the presence of DCPA than that in the serum treatment alone (Fig. 6), suggesting that DCPA treatment inhibits HIF-1α protein expression through the proteosomal degradation pathway. Although the signaling pathways that control HIF-1α expression mediated by DCPA are still unknown, it is possible that HIF-1α expression is inhibited DCPA via the inhibition of PI3K signaling pathway, which activates AKT [19]. It is known that HIF-1α expression is inhibited by PI3K inhibitors and the tumor suppressor PTEN [2,10]. DCPA may mimic PTEN to inhibit PI3K signaling and thus, HIF-1α expression. AKT is also known to activate the transcription factor NF-κB and DCPA down regulates NF-κB activation [11]. DCPA also selectively inhibits proinflammatory cytokines as well as IL-2 by down regulating transcription [11-16]. Hypoxia condition is commonly observed during tumor growth. To determine whether DCPA treatment affected hypoxia-induced HIF-1α expression, the cells were cultured in the absence or presence of 1% O_2 _condition. DCPA treatment greatly inhibited hypoxia-induced HIF-1α protein level (Fig. 7), suggesting that DCPA commonly inhibits certain signaling pathways for regulating growth factor- and hypoxia-induced HIF-1α expression. It is also possible that there may be a connection between inhibition of HIF-1α expression and inhibition of cell migration, which would be a very interesting topic for the future study. These results indicate that DCPA may inhibit prostate cancer cell migration, proliferation, and HIF-1α expression through multiple signaling pathways.

## Conclusion

In conclusion, these results demonstrated that DCPA inhibits cell migration, proliferation, and HIF-1α expression in prostate cancer cells. To further understand the molecular mechanism of DCPA in decreasing HIF-1α expression in these cells, we found that 1) DCPA specifically inhibits the induction of HIF-1α expression by serum, 2) expression of HIF-1β is not altered by the addition of serum or DCPA, 3) the effect of DCPA on HIF-1α is not due to the non-specific effect in the cells, 4) DCPA treatment decreased HIF-1α expression by affecting its stability through the proteasomal degradation pathway, and 5) DCPA also inhibited hypoxia-induced HIF-1α expression in human prostate cancer cells.

## Abbreviations

Abbreviations: DCPA, n-3, 4-dichlorophenyl propanamide; FBS, fetal bovine serum; HIF-1, Hypoxia-inducible factor 1; IL, Interleukin; IGF-I, insulin-like growth factor I; MEM, Minimum Essential Medium; PI3K, phosphatidylinositol 3-kinase; SFM, serum-free medium; VEGF, vascular endothelial growth factor.

## Competing interests

The author(s) declare that they have no competing interests.

## Authors' contributions

BHJ performed initial experiments in this study and the overall supervision of the project, and drafted the manuscript; LZL acquired the data for Figs. 5, 6, 7; RS and DCF participated in the design of the study and manuscript preparation; and JBB participated in the design of the study, data interpretation and manuscript preparation. All authors read and approved the final version of the manuscript.

## Pre-publication history

The pre-publication history for this paper can be accessed here:



## References

[B1] Jiang BH, Agani F, Passaniti A, Semenza GL (1997). V-SRC induces expression of hypoxia-inducible factor 1 (HIF-1) and transcription of genes encoding vascular endothelial growth factor and enolase 1: involvement of HIF-1 in tumor progression. Cancer Res.

[B2] Jiang BH, Rue E, Wang GL, Roe R, Semenza GL (1996). Dimerization, DNA binding, and transactivation properties of hypoxia-inducible factor 1. J Biol Chem.

[B3] Semenza GL (2001). HIF-1, O2, and the 3 PHDs: How Animal Cells Signal Hypoxia to the Nucleus. Cell.

[B4] Zhong H, De Marzo AM, Laughner E, Lim M, Hilton DA, Zagzag D, Buechler P, Isaacs WB, Semenza GL, Simons JW (1999). Overexpression of hypoxia-inducible factor 1alpha in common human cancers and their metastases. Cancer Res.

[B5] Jiang BH, Jiang G, Zheng JZ, Lu Z, Hunter T, Vogt PK (2001). Phosphatidylinositol 3-kinase signaling controls levels of hypoxia-inducible factor 1. Cell Growth Differ.

[B6] Frost LW, Neeley YX, Schafer R, Gibson LF, Barnett JB (2001). Propanil inhibits tumor necrosis factor-a production by reducing nuclear levels of the transcription factor, nuclear factor-kappaB in the macrophage cell line, IC-21.. Toxicol Appl Pharmacol.

[B7] Xie YC, Schafer R, Barnett JB (1997). The immunomodulatory effects of the herbicide propanil on murine macrophage interleukin-6 and tumor necrosis factor-alpha production.. Toxicol Appl Pharmacol.

[B8] Zhao W, Schafer R, Cuff CF, Gandy J, Barnett JB (1995). Changes in Primary and Secondary Lymphoid Organ T-Cell Subpopulations Resulting from Acute In Vivo Exposure to Propanil. J Toxicol Environmental Health.

[B9] Jiang BH, Semenza GL, Bauer C, Marti HH (1996). Hypoxia-inducible factor 1 levels vary exponentially over a physiologically relevant range of O2 tension. Am J Physiol.

[B10] Maxwell PH, Dachs GU, Gleadle JM, Nicholls LG, Harris AL, Stratford IJ, Hankinson O, Pugh CW, Ratcliffe PJ (1997). Hypoxia-inducible factor-1 modulates gene expression in solid tumors and influences both angiogenesis and tumor growth. Proc Natl Acad Sci U S A.

[B11] Zhong H, Chiles K, Feldser D, Laughner E, Hanrahan C, Georgescu MM, Simons JW, Semenza GL (2000). Modulation of hypoxia-inducible factor 1alpha expression by the epidermal growth factor/phosphatidylinositol 3-kinase/PTEN/AKT/FRAP pathway in human prostate cancer cells: implications for tumor angiogenesis and therapeutics. Cancer Res.

[B12] Laughner E, Taghavi P, Chiles K, Mahon PC, Semenza GL (2001). HER2 (neu) signaling increases the rate of hypoxia-inducible factor 1alpha (HIF-1alpha) synthesis: novel mechanism for HIF-1-mediated vascular endothelial growth factor expression. Mol Cell Biol.

[B13] Huang LE, Gu J, Schau M, Bunn HF (1998). Regulation of hypoxia-inducible factor 1alpha is mediated by an O2-dependent degradation domain via the ubiquitin-proteasome pathway. Proc Natl Acad Sci U S A.

[B14] Salceda S, Caro J (1997). Hypoxia-inducible factor 1alpha (HIF-1alpha) protein is rapidly degraded by the ubiquitin-proteasome system under normoxic conditions. Its stabilization by hypoxia depends on redox-induced changes. J Biol Chem.

[B15] Xie YC, Schafer R, Barnett JB (1997). Inhibitory effect of 3,4-dichloro-propionaniline on cytokine production by macrophages is associated with LPS-mediated signal transduction.. J Leukoc Biol.

[B16] Zhao W, Schafer R, Barnett JB (1996). Cytokine production by C57Bl/6 mouse spleen cells is selectively reduced by exposure to propanil. J Toxicol Environ Health.

[B17] Zhao W, Schafer R, Barnett JB (1999). Propanil affects transcriptional and posttranscriptional regulation of IL-2 expression in activated EL-4 cells. Toxicol Appl Pharmacol.

